# Blended clustering energy efficient routing and PUF based authentication in IoT enabled smart agriculture systems

**DOI:** 10.1038/s41598-025-07917-3

**Published:** 2025-07-09

**Authors:** Senthil Kumar Chandrasekaran, Vijay Anand Rajasekaran

**Affiliations:** Department of Information Technology, School of Computer Science Engineering and Information Systems (SCORE), Vellore Institute of Technology, Vellore, India

**Keywords:** IoT-based smart agriculture systems, Energy, BCEER, PUF-based authentication, Homomorphic encryption, Sign function, Computer science, Information technology

## Abstract

This study presents a simulation-based approach to improving energy efficiency and security in IoT-based smart agriculture systems. The suggested architecture combines a new Blended Clustering Energy Efficient Routing (BCEER) protocol with an authentication mechanism based on Physical Unclonable Functions (PUFs) to respond to the main issues in resource-limited, sensor-oriented settings. BCEER maximizes cluster head selection using adaptive energy thresholds to minimize communication overhead and ensure extended network lifetime. Moreover, authentication based on PUF provides hardware-level identity authentication through challenge-response pairs, without demanding heavy cryptography computation, to improve security. For safe signal processing, polynomial approximation techniques were analyzed for sign function approximation, and error analysis indicated improving accuracy as the approximation degree grows. The findings show that the hybrid method enhances energy distribution, enhances authentication, and provides computationally efficient signal processing, making the system relevant for efficient and secure large-scale deployment in intelligent farming IoT networks.

## Introduction

The quick improvement of Internet of Things (IoT) development has set off a historic disturbance in agriculture, completing the ascent of IoT-based Smart Water framework Systems^1^. This significant impact in context suggests a take-off from ordinary developing procedures, handling interconnected contraptions, undeniable level data assessment, and motorization to rename water framework practices. At its essence, IoT-based Smart Water framework Systems epitomize the mix of cutting-edge advancements with age-old cultivating customs, promising to lift proficiency, productivity, and sustainability in rural water framework processes. Fundamentally, IoT mix in smart water framework systems spreads out an interconnected climate where parts like soil, yields, devices, and environmental conditions impeccably give and exchange continuous data^2^. This interconnectedness empowers farmers and accomplices with huge encounters, empowering precision water framework practices. Unlike evident and experiential data in standard development, smart plant systems filled by IoT present a new era of data-driven development.

The degree of potential IoT applications in smart country game plans wraps around a lot of interconnected devices, including sensors, actuators, drones, and electronic equipment. These conclusively arranged contraptions gather grouped data centers, huge for efficient water framework management^3^. Soil sensors screen sogginess levels and supplement content, drones get raised imagery for crop prosperity examination, and mechanized water framework systems precisely pass water to express districts at ideal times. This mechanical mix updates resource use as well as cutoff points’ normal impact, controlling provincial practices towards sustainability and eco-insightful systems^4^. In any case, the fundamental test in the space of Heterogeneous IoT inside smart agriculture lies in spreading out a secure, reliable, and naturally conscious environment for all devices. The different thoughts of these contraptions, wrapping fluctuating hardware, software, and communication show, present complexities in interoperability and security. Watching out for this challenge requires the improvement of a generous and secure groundwork fit for managing the various intricacies inherent in IoT systems, while also zeroing in on energy efficiency, a fundamental idea as a result of the reliance of various IoT devices on confined energy resources.

Efficient energy routing and lively well-being endeavours emerge as key help focuses in supporting IoT-based Smart Water framework Systems^5^. The joining of energy-efficient routing shows works on utilitarian efficiency, directing energy resources and extending device lifetimes, agreeing with sustainability objectives. Meanwhile, PUF-based authentication gives a gear laid out and highly secure strategy for device recognition proof, ensuring strong security in IoT communications, particularly in sending delicate rustic data. The mix of energy-efficient routing instruments and PUF-based authentication inside smart water framework systems indicates a huge shift in key areas of strength towards secure IoT ecosystems modified for momentum agriculture^6^. While serving undeniable functionalities, these integrated parts are innately associated, adding to the creation of efficient, secure, and dependable associations major for improving water framework practices and ensuring cultivating sustainability.

The association of energy-efficient routing and PUF-based authentication tends to an adjustment of viewpoint in arranging solid and viable IoT ecosystems. These two sections, while keeping an eye on different pieces of IoT convenience, are characteristically interconnected in their endeavour to make efficient, secure, and strong associations. Energy-efficient routing shows that, by propelling data transmission and social events, it directly adds to energy resource insurance^7^. Contraptions chipping away at confined energy stores could extend their useful lives, diminishing the frequency with which batteries ought to be replaced or recharged. These lines up with regular objectives as well as work on the overall dependability of IoT processes. All the while, PUF-based authentication supports the security surface of the IoT association. As contraptions convey and exchange tricky information, the strong and stand-out identifiers given by PUFs ensure careful device conspicuous proof and endorsement. This hardware-based authentication is extremely effective against typical computerized risks, for instance, cloning, replay attacks, and unauthorized access tries, providing a safeguarded foundation for IoT transactions^8^.

## Related work

Countless investigations dive into heterogeneous IoT systems, using various methodologies. These wrap computer-based intelligence for data examination, undeniable level encryption for a security overhaul, hardware smoothing out, and innovative communication tools, all adding to enhanced execution and utility across a scope of contraption associations. A couple of noteworthy responsibilities are inspected.

Viswanathan^9^ presents a low-energy, secure routing strategy for IoT in heterogeneous Distant Sensor Associations (WSNs). The proposed plot beats existing shows by using Multipath Association Routing Show (MLRP) over secure associations, further developing throughput, energy efficiency, end-to-end latency, network lifetime, and data storage limit. Besides, Cross variety of Youth (H-High schooler) is utilized for enhanced energy smoothing out and stack changing, committing to movements in IoT applications inside various WSN conditions.

Hu et al.^10^ presents TBSEER, a unique trust-based, energy-efficient routing framework that highlights restricting security threats in WSNs. TBSEER registers total trust regard, solidifying versatile prompt, roaming, and energy trust values to shield against various risks. It uses a flexible discipline part and a capriciousness factor for quick conspicuous proof of noxious center points.

Nadu et al.^11^ presents EAOCSR, a sharp strategy solidifying efficient clustering, dependable routing, and transmission to redesign energy economy and association lifetime. By using unbalanced clustering and trust-based secure routing, the proposed approach beats existing approaches concerning energy usage, throughput, network lifetime, adequacy, and security.

Lakshmanan^12^ presents mod-EHTARA, a strong routing solution that further develops energy viability and the future center point. Joining the Association Lifetime model with EHTARA works with the ID of secure routing ways based on exhaustive cost norms. The assessment develops its certification to immense data arrangement at the base station, utilizing the Aide Reduction plan and stacked autoencoders arranged by the original Flexible E-Bat procedure. Mod-EHTARA shows unmatched execution, achieving maximal energy efficiency of 0.9855.

Shwetha et al.^13^ presents CBE-ECR, a technique for securing data on the board and transmission utilizing Code-Based Encryption with Energy Use Routing. CBE-ECR overhauls data security, limits unapproved access, and efficiently controls network future through keyed-hash message authentication. It shows a prominent 96.17% improvement in data on the board feasibility and a 21.11% reduction in energy usage, diverged from renowned procedures.

Udayaprasad et al. ^14^ research aims to increase distributed compute-dependent Software Defined Network (SDN) with high-level IoT. The research proposes an energy-efficient routing technique for large-scale IoT networks using Genetic Algorithm, Particle Swarm Optimization, and Artificial Bee Colony. The proposed routing minimizes energy dissemination and achieves enhanced energy balance, improving network lifetime and energy usage.

Yalli et al.^15^ reviews the security challenges of IoT systems for authentication, analyzing strengths, weaknesses, threats, and attacks. It examines IoT-compatible protocols, enabling technologies, and countermeasures. The study uses the PRISMA methodology to compare advances in securing IoT devices and highlights the need for integrating authentication models with compatible protocols.

The rapid expansion of IoT in healthcare necessitates robust security measures^16^. A secure, resource-efficient cloud-enabled authentication mechanism is introduced, reducing computational and communication overheads, improving system efficiency, and ensuring resilience against cyberattacks. This robust mechanism is crucial for protecting sensitive medical information.

The Internet of Medical Things (IoMT)^17^ is a promising framework for telemedicine, but security threats and authentication issues persist. A lightweight authentication protocol using Physically Unclonable Function (PUF) addresses these challenges. This protocol outperforms similar schemes in security and efficiency, providing flexibility for different scenarios and working conditions.

**Table 1:** Comparison of secure and trust-based routing protocols.

Reference	Acronyms	Security model	Energy consideration	Network overhead cost	Applicability	Defence against internal attacks
^18^	SHOP	Symmetric and asymmetric cryptosystems	Yes	High	Limited	No
^19^	HCBS	Hybrid cryptography	No	High	Limited	No
^20^	ECCSRA	Cyclic group-based elliptic curve cryptography	No	High	Limited	No
^21^	TrufiX	Trust-based fuzzy implicit cross-layer	Yes	Moderate	Widespread	Yes
^22^	ESCORT	Weighted sum of trust, energy, and hop counts	Yes	Moderate	Limited	Yes
^23^	TESRP	Weighted sum of trust, energy, and hop counts	Yes	Moderate	Limited	Yes
^24^	TSRRM	Trust degree && QoS metrics	Yes	Moderate	Limited	Yes
^25^	SQEER	Trust model && authentication technique with a key	Yes	Moderate	Limited	Yes
^24^	LTMS	Trust management based on the binomial distribution	Yes	Moderate	Limited	Yes

Table 1 represents the comparison of secure and trust-based routing protocols. These works overall add to pushing the field of IoT-enabled smart agriculture systems, providing pieces of information into energy-efficient routing techniques, enthusiastic security parts, and trust-based approaches central for smoothing out water framework practices and ensuring green sustainability.

The core problem addressed in this research is the lack of an integrated, energy-efficient, and secure communication framework tailored for IoT-enabled smart agriculture systems, particularly in resource-constrained rural environments. Existing protocols often optimize either energy efficiency or security, but not both, leading to trade-offs that limit practical deployment in agricultural settings. This study proposes a novel synergy of Blended Clustering-Based Energy-Efficient Routing (BCEER) and Physical Unclonable Function (PUF)-based hardware authentication, a combination not previously explored in this domain. The novelty lies in: (1) integrating dynamic cluster head selection based on residual energy to prolong network lifetime; (2) introducing hardware-level, clone-resistant authentication via PUFs to secure sensor communications; and (3) applying high-precision polynomial approximation to optimize the sign function involved in signal processing, enhancing computational reliability. Together, these innovations provide a unified solution that balances scalability, sustainability, and security, addressing critical gaps in the deployment of robust IoT infrastructures for precision agriculture.

## Problem definition

In the location of the Internet of Things (IoT), the setting of a Smart Water framework Structure depicts a climate portrayed by viewpoints H (height) and W (width), prepared for accommodating n center points, definitively dispersed across the country locale. Right away, all centers have unclear energy early phases (E_initial). Along these lines, the energy levels of every center point go through strong modifications driven by individual center lead, network direct, and winning normal conditions.

Additionally, inside this structure, the energy potential gains of every center are invigorated with each transmission, subject to the specific approach to the acting of the center point and the normal state of the water framework association. To strengthen the overall security of Smart Water framework Systems, encryption apportions are implemented to ensure the protection and trustworthiness of sent data, as needed, supporting the general security position of the structure. Additionally, invaluable strategies like PUF (Real Unclonable Capacity)- based authentication are solidified to develop the security framework. PUFs impact the characteristic genuine changes in gear parts to equip unique, unclonable characters for each IoT device inside the water framework structure. This development overhauls the security of the structure by introducing PUF-based authentication, which mitigates unapproved access, ensuring a more secure and robust communication environment for IoT devices within the space of smart water frameworks in agriculture^15^.

### Methodology

#### Energy consumption model

The energy use model delineated in this section frames the energy components inside the proposed BCEER (BCEER-Enhanced Energy-Efficient Routing). This model serves as a basic design for understanding and further developing energy use in IoT (Internet of Things) associations, particularly in the context of smart agriculture systems^26,27^.

**Fig. 1:**
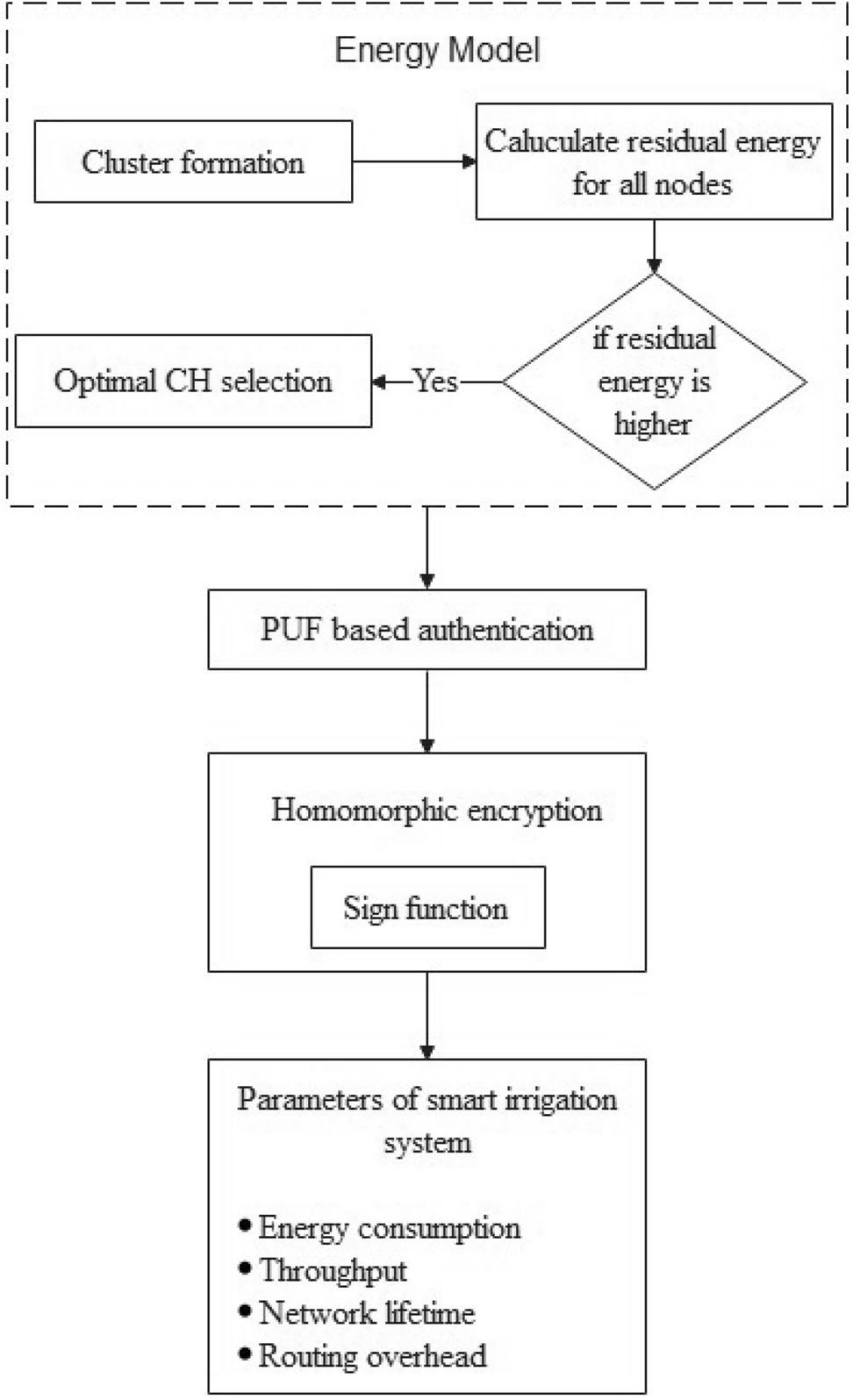
Flow chart for the proposed method.

In standard IoT systems, for instance, those used in smart agriculture, energy use is an essential concern in light of the reliance on battery-powered sensor centers^28,29^. Efficient organization of energy resources is fundamental to ensure the association’s future and ensure persistent movement. The BCEER shows watches out for this test by introducing a broad energy use model that records various factors affecting energy use in IoT associations. The flow chart of the proposed model is mentioned in Fig. 1.

*Energy consumption components*:

The energy used inside the BCEER show is made from a couple of key parts, each adding to the overall energy utilization of the system. These parts include:

*Cluster head energy consumption (E_CH)*:

Cluster Heads (CHs) play a critical part in IoT associations, filling in as go-betweens between sensor nodes and base stations. The energy use of CHs wraps a couple of factors, including:

*Transmission energy (E_elec):*

The energy consumed by a CH while taking care of and imparting data to the base station. This energy use is compared to how much data is being conveyed.

*Data aggregation energy (E_DA):*

The energy consumed by a CH during data assortment, where overabundance or redundant data from sensor centers is converged before transmission to the base station.

*Free space path loss (ε_fs):*

The energy is scattered as a result of the given narrowing of distance in free space. This loss is inversely proportional to the square of the distance between the CH and the base station (d_toBS^2).

The total energy consumption of a CH (E_CH) is determined by the sum of these factors, as expressed by the equation:

1 $$\:ECH=(\frac{N}{K}-1)\cdot\:m\cdot\:{E}_{elec}+\frac{N}{K}\cdot\:m\cdot\:{E}_{DA}+m\cdot\:{E}_{elec}+m\cdot\:{\epsilon\:}_{fs}\cdot\:{d}_{toBS}^{2}$$

Where:

- *N* is the total number of nodes in the IoT network,
- *K* is the total number of clusters in each round,
- *m* is the average number of cluster nodes,
- *E*_*elec*_​ is the energy consumed by a CH while processing a one-bit message,
- *E*_*DA*_ is the energy consumed during data aggregation,
- *ε*_*fs*_​ is the free space path loss coefficient,
- *d*_*toBS*_​ is the distance between the CH and the base station.

*Non-cluster head energy consumption (E_nonCH)*:

Non-cluster heads (non-CHs) represent the remaining sensor nodes within each cluster. These centers add to energy usage essentially through data transmission and social affairs. The energy usage of non-CHs is influenced by factors, for instance,

*Transmission energy (E_elec):*

Like CHs, non-CHs consume energy during data transmission.

*Free Space Path Loss (ε_fs):*

Energy dispersal on account of the given narrowing up distance is not settled by the square of the distance between the center point and its CH (d_toCH^2).

The total energy consumption of non-CHs (E_nonCH) is given by the equation:

2 $$\:{E}_{nonCH}\:=\:m\cdot\:{E}_{elec}+m\cdot\:{\epsilon\:}_{fs}\cdot\:{d}_{toBS}^{2}$$

Where:

- d_toCH_​ is the distance between an individual sensor node and its CH.

*Cluster energy consumption (E_cluster)*:

The energy use of each bundle inside the IoT network is how much energy is consumed by its CH and non-CHs. Equation (5) expresses the total energy consumption of a cluster (E_cluster):

3 $$\:{E}_{cluster}\:=\:{E}_{CH}+(\frac{N}{k}-1)\cdot\:{E}_{nonCH}\approx\:{E}_{CH}+\frac{N}{k}\cdot\:{E}_{nonCH}$$

Where:

- *k* is the total number of clusters,
- $\:\frac{N}{k}$ Represents the average number of cluster nodes.

*Round energy consumption (E_round)*:

The energy use of the BCEER shows all through each cycle (round) is how much energy is consumed by all bundles. Equation 4 provides an expression for the total energy consumption of the BCEER protocol (E_round):

4 $$\:{E}_{round}=k\cdot\:{E}_{cluster}=m\cdot\:(2N{E}_{elec}+{NE}_{DA}+{k\epsilon\:}_{fs}\cdot\:{d}_{toBS}^{2}+N{\epsilon\:f}_{s}\cdot\:{d}_{toBS}^{2})$$

*Optimal number of cluster heads (k_opt)*:

The ideal number of Gathering Heads (CHs) in the BCEER is not altogether firmly established by Condition 5, where *k*_*opt*_ addresses the ideal number of CHs based on the association limits:

5 $$\:{k\:}_{opt}=\:\sqrt{\frac{N}{2\pi\:}}\cdot\:\frac{L}{{d}_{toBS}}$$

Where:

- L is the area of the dispersed node region.

The energy use model of the BCEER gives an exhaustive framework for looking at and further developing energy utilization in IoT associations. By addressing components like transmission energy, data gathering energy, and way hardship, the model enables the arrangement of energy-efficient routing schemes tailored to the specific needs of smart agriculture systems. In addition, the confirmation of the ideal number of Gathering Heads works with the efficient task of resources, further improving the life expectancy and sustainability of IoT associations in agrarian circumstances.

#### Energy threshold for the selection of cluster heads

In IoT (Internet of Things) associations, the decision of Bundle Heads (CHs) accepts a urgent part in smoothing out energy utilization and drawing out the association’s future. The Energy Edge instrument introduced inside the BCEER (BCEER-Enhanced Energy-Efficient Routing) show means to change energy usage among sensor centres and assurance the assurance of CHs with satisfactory leftover energy. This part gives a point-by-point explanation of as far as possible estimation and its ideas for CH assurance.

Ordinary CH assurance shows, similar to Deplete (Low Energy Flexible Clustering Request), often experience the adverse impacts of limitations in ensuring the ongoing waiting energy of CHs. Unpredictable assurance of CHs dismissing their energy levels could provoke less than ideal utilization of energy resources, resulting in network fragility and reduced life expectancy. As far as possible, estimation watches out for this test by dynamically changing the probability of picking CHs based on their energy levels.

*Energy adjustment setting*:

The Energy Change Setting inside the Energy Edge computation chooses the probability of picking a centre as a CH based on its continuous excess energy and association limits. The probability (*pi*) of picking a centre point (*si*) as a CH is resolved using Condition 6:

6 $$\:{p\:}_{i}=\:\frac{p\cdot\:{s}_{i}\cdot\:{E}_{r}^{i}\cdot\:{Ei}_{i}}{\:{E}_{t}\cdot\:{E}_{a}}$$

Where:

- *p* is the probability of picking the best CH,
- $\:{E}_{r}^{i}$ Is the current residual energy of node *si*,
- *E*_*i*_​ is the initial energy of node *si*,
- *E*_*t*_​ is the network’s total capacity,
- *E*_*a*_​ is the total energy consumed by all sensor nodes.

The Energy Change Setting calculation changes the likelihood of hub choice based on its energy status compared with the organization’s absolute energy limit. Hubs with higher leftover energy levels are bound to be chosen as CHs, advancing energy balance inside the organization.

*Grand threshold calculation*:

As far as possible assessment sums individual centre energy edges to choose the capability of centre points for CH assurance. Condition 7 presents the assessment of the average energy level () expected for CH assurance:

7 $$\:{E}_{a}\:=\:\:\frac{{E}_{t}\cdot\:\left(\frac{1-r}{{r}_{max}}\right)}{{s}_{i}}$$

Where:

- r is the current phase of the BCEER protocol,
- r_max​_ is the maximum number of phases,
- s_i_​ is the node.

The Incomparable Edge computation registers the common energy level expected for centre point decision as CHs, ensuring a nice spread of energy usage across the association.

The Energy Edge framework inside the BCEER shows redesigns CH assurance by considering the ongoing extra energy of center points and capably changing the probability of center point assurance based on their energy levels. By propelling energy balance and postponing the future of CHs, as far as possible, estimation adds to the overall sustainability and efficiency of IoT networks in smart agriculture systems.

#### Physical unclonable function (PUF) based authentication

Certified Unclonable Limits (PUFs) provide a brutal method for device verification and information integrity checks about IoT (Web of Things) security. This part makes sense of how PUF-based validation functions and how significant it is in IoT-empowered intelligent farming frameworks, zeroing in on how it further develops security and capability^30^.

**Fig. 2:**
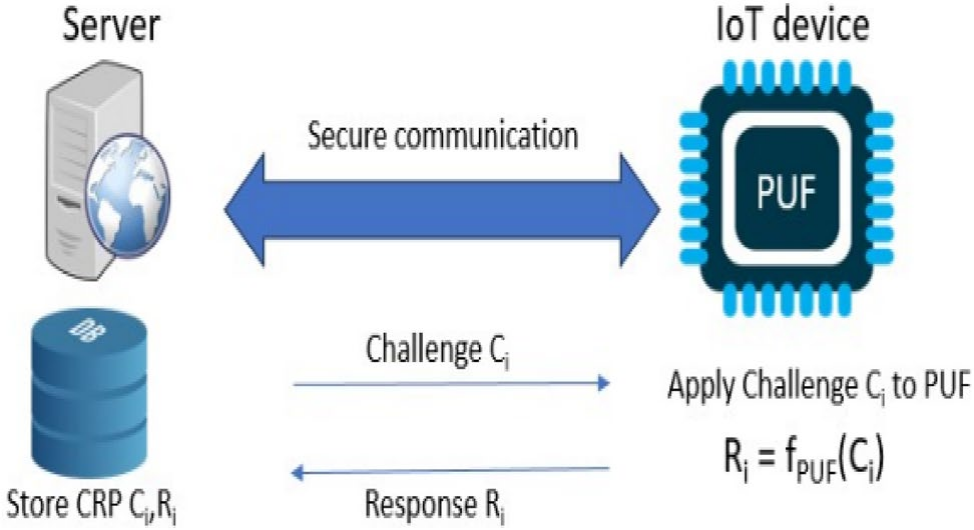
Authentication scheme using PUF CRPs.

PUFs exploit ordinary certifiable game plans in gear parts to convey unique and unclonable identifiers for IoT devices. These identifiers are likely to be cryptographic keys or validation tokens, supporting the IoT affiliations’ security strategy. PUF-based affirmation gives a reliable instrument for checking the individual and integrity of contraptions, planning gambles related to unapproved access and data evolving^31,32^. Schematic representation of Authentication using PUF CRPs described in Fig. 2.

*Dynamic energy management*:

Dynamic energy on the board is significant to the movement of IoT devices inside smart farming systems. Based on network conditions, organic elements, and focus direction, energy values at each middle point fluctuate. This strong energy that the trailblazers ensure ideal contraption execution and adds to the future of the IoT climate.

*Role of PUFs in authentication*:

PUFs make unprecedented mechanized fingerprints considering the real characteristics of gear parts. These fingerprints serve as device identifiers, engaging secure approval and communication inside IoT associations. By using the inherent variability of gear, the PUF-based check gives strong protection from cloning and emulation attacks^33^.

*Implementation of PUFs*:

The execution of PUF-based check incorporates getting and taking care of the original responses made by each contraption’s hardware. During correspondence meetings, these reactions are used to validate devices to ensure that the primary approved devices can reach the organization. PUFs redesign the security of IoT structures by giving a gear-based affirmation framework that is impenetrable to changing and duplication.

*Symbiotic relationship with energy management*:

The mix of PUF-based approval with dynamic energy on the board makes an agreeable relationship that works on the overall security and capacity of IoT affiliations. Compelling energy use takes out contraption future and down to earth steadfastness, while PUF checks safeguard against security threats and unapproved access. This mix ensures a versatile and secure correspondence environment for IoT devices in smart farming structures.

PUF-based check offers strong regions for a contraption affirmation and data legitimacy confirmation in IoT-engaged splendid cultivation systems. By including genuine blends in gear parts, PUFs give remarkable identifiers that further encourage security and back off bets related to unapproved access. They set out a reasonable compromise of PUF-based confirmation with dynamic energy management, making a synergistic method that arrangements with both security and capacity in IoT affiliations, adding to the sensitivity and overcoming nature of smart cultivation structures.

#### Physical unclonable function (PUF)

Physical unclonable functions (PUFs) are cryptographic features that exploit the normal physical variations present in hardware parts to make remarkable and unclonable identifiers^34^. These identifiers serve as cryptographic keys or authentication tokens, providing a protected technique for verifying the character and integrity of devices within IoT associations. PUFs offer an energetic defense against cloning and emulate attacks, making them principal parts of wellbeing models in various spaces, including IoT-enabled smart agriculture systems^30^.

PUFs were made to address the creation prerequisite for secure authentication parts in IoT and other embedded systems. Standard cryptographic systems rely upon secret keys set aside in software or hardware, making them vulnerable to attacks like dismantling and key extraction. PUFs offer a hardware based elective that utilization physical properties novel to each device, ensuring that cryptographic keys can’t be rehashed or meddled with.

*Principle of operation*:

The movement of a PUF is based on the clever physical characteristics of hardware parts, such as gathering assortments, material defects, and cycle prompted irregularities. These assortments achieve unpretentious differentiations in contraption direct, which can be exploited to make device unequivocal identifiers^35^. PUFs consistently involve a test response framework, where a test is input into the device, and the contraption makes an exceptional response based on its physical properties. The response fills in as a mechanized finger impression or cryptographic key that is extraordinary to each contraption^36^.

*Types of PUFs*:

There are several types of PUFs, each exploiting different physical phenomena to generate unique identifiers. Some common types of PUFs include:

Arbiter PUF: Utilizes the delay differences in signal propagation through symmetric paths to generate responses^37^.

Ring oscillator PUF: Relies on the variations in ring oscillator frequencies due to process variations to generate responses.

SRAM PUF: Exploits the process-induced variations in SRAM cell stability to produce unique responses.

Each type of PUF has its own advantages and limitations, and the choice of PUF depends on factors such as application requirements and security considerations.

*Security considerations*:

PUFs offer a couple of safety benefits over standard cryptographic techniques. Since PUF-based keys are gotten from physical properties of the device, they are impenetrable to dissecting and cloning attacks^38^. Moreover, PUF responses are created on-demand and are not placed away wherever on the device, making them less frail against physical attacks. In any case, PUFs are not immune to all attacks, and wary arrangement and execution are mean a lot to ensure their security^39,40^.

*Integration with IoT systems*:

In IoT-enabled smart agriculture systems, PUFs expect an enormous part in guaranteeing the security and validity of information conferred between contraptions. By giving uncommon contraption identifiers and cryptographic keys, PUFs connect with secure authentication and correspondence, even in asset obliged conditions. PUF-based authentication instruments can be incorporated into existing IoT shows and models, giving a liberal protection from unapproved access and information changing^41,42^.

PUFs offer a stuff-based answer for gadget authentication and information security in IoT-enabled smart agriculture systems. By utilizing the special physical properties of equipment parts, PUFs give serious areas of strength for a for insisting gadget character and guaranteeing information uprightness. As IoT keeps on copying in agriculture and different undertakings, the joining of PUF-based authentication systems will turn out to be consistently basic in safeguarding IoT ecosystems against security chances.

#### Homomorphic encryption

Homomorphic encryption is a cryptographic technique that licenses computations to be performed on mixed data without unravelling it. This property enables security protecting data dealing with, where tricky information can be chipped away at while exceptional encoded, hence safeguarding it from unapproved access. Homomorphic encryption has basic applications in secure data re-examining, conveyed processing, and assurance shielding computer-based intelligence.

Homomorphic encryption was familiar with address the trial of securely taking care of fragile data while protecting security. Standard encryption methods anticipate that data should be unscrambled preceding performing computations, introducing it to potential security bets. Homomorphic encryption engages estimations to be performed clearly on encoded data, ensuring that plaintext data stays characterized all through the computation cycle.

*Principle of operation*:

The standard of homomorphic encryption is based on mathematical undertakings that defend the numerical plan of the plaintext data when mixed. Specifically, homomorphic encryption plans are expected to help explicit logarithmic exercises, similar to extension and duplication, on ciphertexts, which contrast with playing out comparable strategy on the plaintexts. This property grants estimations to be finished on mixed data without uncovering the crucial plaintext.

*Types of homomorphic encryption*:

There are several types of homomorphic encryption schemes, each offering different levels of computational capabilities:

Partially homomorphic encryption (PHE): Supports either addition or multiplication operations on ciphertexts, but not both.

Somewhat homomorphic encryption (SHE): Supports a limited number of additions and multiplications on ciphertexts, but with restricted computational depth.

Fully homomorphic encryption (FHE): Maintains sporadic mixes of extension and increment strategy on ciphertexts, engaging boundless computational profundity.

Totally homomorphic encryption is the greatest sort of homomorphic encryption, allowing complex estimations to be performed on encoded data without requirements. In any case, FHE schemes consistently cause higher computational above stood out from PHE and SHE plots.

*Security considerations*:

The security of homomorphic encryption schemes relies on the hardness of certain mathematical problems, such as the Integer Factorization Problem (IFP) or the Learning with Errors (LWE) problem. The security of a homomorphic encryption scheme is typically analyzed in terms of its resistance to known cryptographic attacks, such as chosen plaintext attacks and ciphertext attacks.

*Applications*:

Homomorphic encryption has diverse applications in various domains:

Secure data outsourcing: Allows sensitive data to be processed by third-party service providers without exposing it to potential security risks.

Privacy-preserving machine learning: Enables machine learning algorithms to operate on encrypted data while preserving the privacy of sensitive information.

Secure cloud computing: Facilitates secure computation on encrypted data in cloud environments, protecting sensitive data from unauthorized access.

*Challenges and future directions*:

Disregarding its promising applications, homomorphic encryption really faces a couple of challenges, including computational above, key organization, and flexibility. Watching out for these hardships will be crucial for the vast gathering of homomorphic encryption in evident applications. Likewise, consistent assessment intends to encourage more capable and rational homomorphic encryption plans with additional created execution and security guarantees.

### Experimental setup

The experimental implementation of the proposed BCEER-enhanced routing and PUF-based authentication framework was carried out MATLAB 2023a, chosen for its flexibility and efficiency in scientific computing. All simulations were executed on a standard Windows 11 system equipped with an Intel Core i7 1065G7 processor and 32 GB RAM. The simulation involved randomized cluster head selection and sensor node distribution across network sizes ranging from 200 to 1000 nodes.

To address numerical precision challenges, particularly in approximating the discontinuous sign function, high-resolution intervals and increasing polynomial degrees were used. Errors were analyzed graphically to ensure consistency between theoretical and actual accuracy. Biases were mitigated by conducting multiple simulation runs under varying parameters and by ensuring randomness in node placement and clustering. Synthetic challenge-response pairs were used in PUF authentication to mimic real-world hardware variability and avoid uniformity bias. Overall, the setup ensured computational robustness, fairness, and repeatability of results in evaluating energy efficiency and security within IoT-enabled smart agriculture systems.

## Results

In this part, we present the total evaluation outcomes of the proposed approaches, including energy-capable coordinating redesigned by the BCEER show and PUF-based approval, inside IoT-enabled wise agribusiness structures. The appraisal integrates alternate points of view, for instance, botch assessment, computation time, and execution estimations of the proposed strategies.

### Error analysis of polynomial approximations

To assess the precision of polynomial approximations in approximating the sign capacity, we coordinated a low-down botch assessment using different blends of polynomials *f*(*x*) and *g*(*x*). The slip-up was evaluated over the stretch [− 1,1] with *xi* = −1+ *i*/1000.

**Fig. 3:**
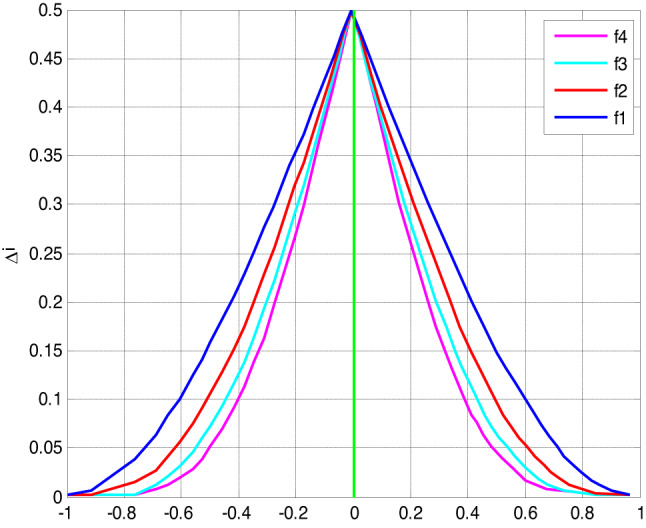
Error of polynomial f_n_ approximating the sign function.

The bumble assessment depicted in Fig. 3 uncovers the association between the level of the polynomial *f*(*x*) and the assessment precision. As the level of the polynomial forms, the screw up lessens, showing additionally created accuracy.

**Fig. 4:**
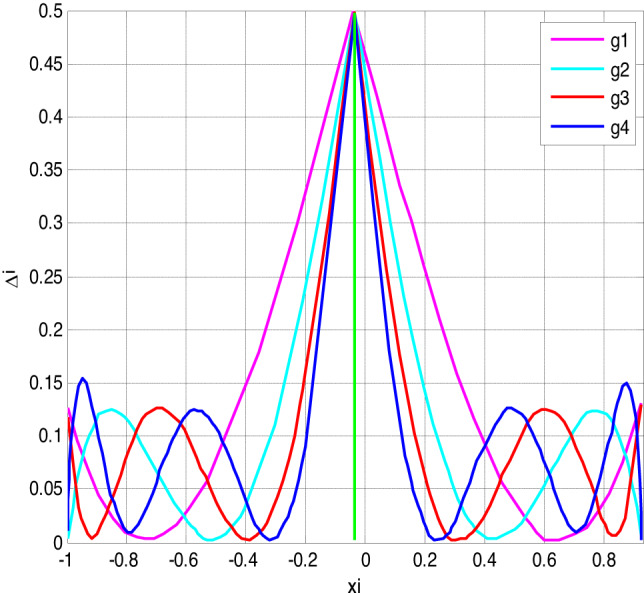
Error of polynomial g_n_ approximating the sign function.

Similarly, Fig. 4 illustrates the error incurred by polynomial *g*(*n*) in approximating the sign function. It demonstrates that as the degree of the polynomial increases, the approximation accuracy improves, albeit with some trade-offs in certain intervals.

**Fig. 5:**
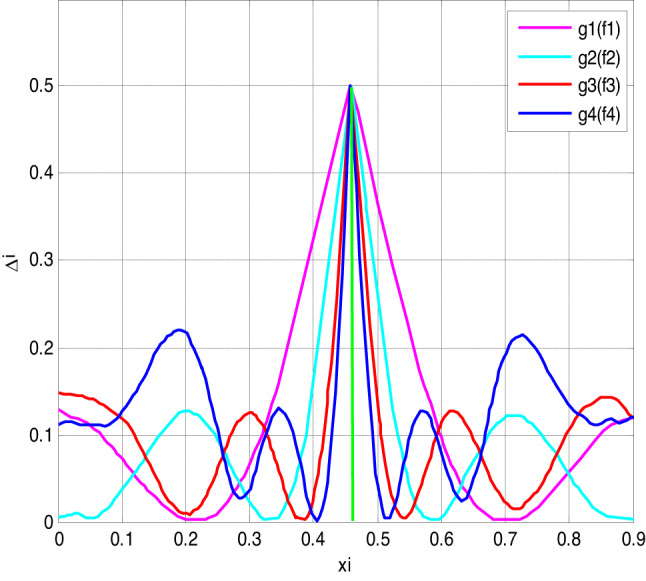
Error of polynomial g_n_ (f_n_) approximating the sign function.

**Fig. 6:**
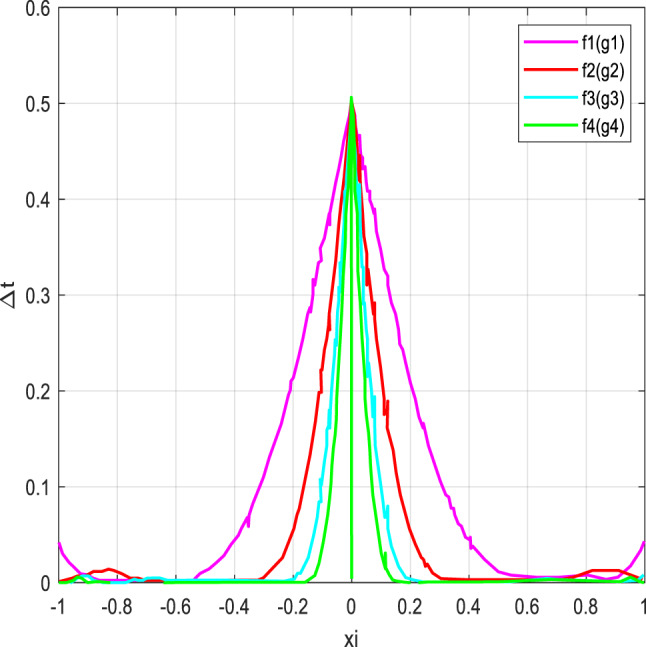
Error of polynomial f_n_ (g_n_) approximating the sign function.

Figures 5 and 6 illustrate the error in approximating the sign function using polynomial functions. Figure 5 represents the error when polynomials g_n_(x) approximate f_n_(x), while Fig. 6 shows the error when polynomials f_n_(x) approximate g_n_(x). The results suggest that while the error remains small in most regions, it peaks significantly near the center, indicating a challenge in achieving precise accuracy at this point. This behavior highlights the limitations of polynomial approximations in capturing the discontinuous nature of the sign function.

Figures 3, 4, 5 and 6 present error analysis for the polynomial approximation of the sign function employed in secure computation, especially in the homomorphic encryption and authentication mechanism. Proper approximation allows for reduced computational overhead, which directly translates to energy saving in resource-limited IoT nodes. By further providing stable and accurate encryption calculation, these approximations maintain the authenticity of the PUF-based authentication, thereby indirectly improving the security of the system.

### Comparison of theoretical and actual accuracy

**Fig. 7:**
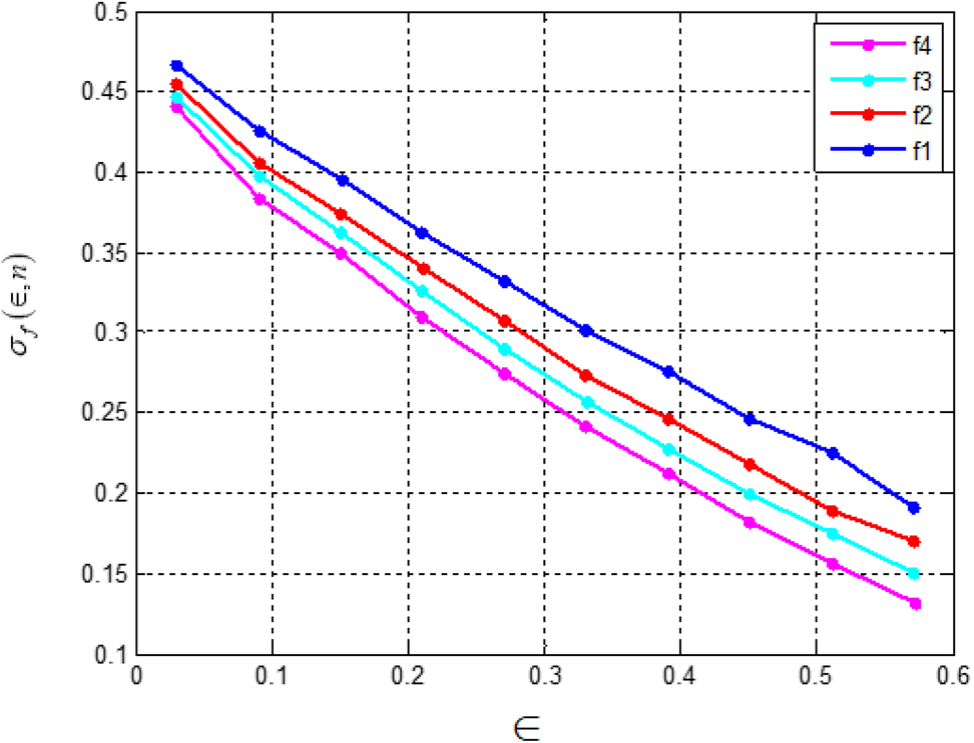
Difference between theoretical accuracy and actual accuracy of the sign function approximation by the polynomials f_n_(*x*) on the interval *x* ∈ [є, 1].

**Fig. 8:**
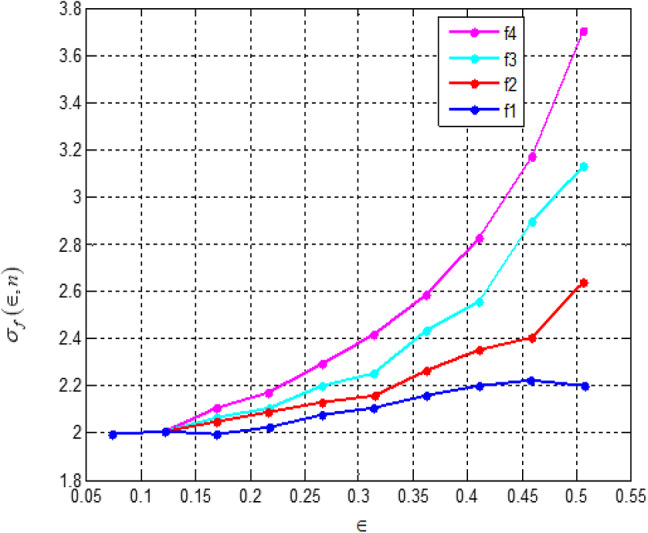
Ratio of theoretical accuracy and actual accuracy of the sign function approximation by the polynomials f_n_(*x*) on the interval *x* ∈ [є, 1].

Figures 7 and 8 present a comparison between the theoretical accuracy and the actual accuracy of the sign function approximation by the polynomials *fn*(*x*). The analysis indicates that while theoretically estimated accuracy is higher, the actual accuracy remains slightly lower, particularly in intervals closer to zero.

**Fig. 9:**
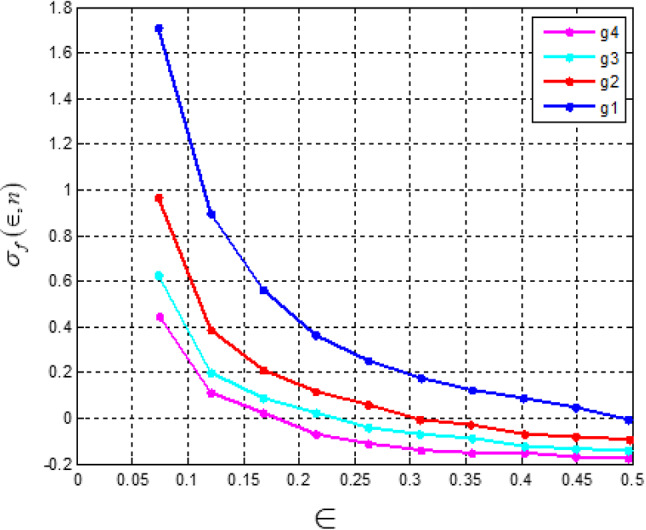
Difference between theoretical accuracy and actual accuracy of the sign function approximation by the polynomials *g*_n_(*x*) on the interval *x* ∈ [є, 1].

**Fig. 10:**
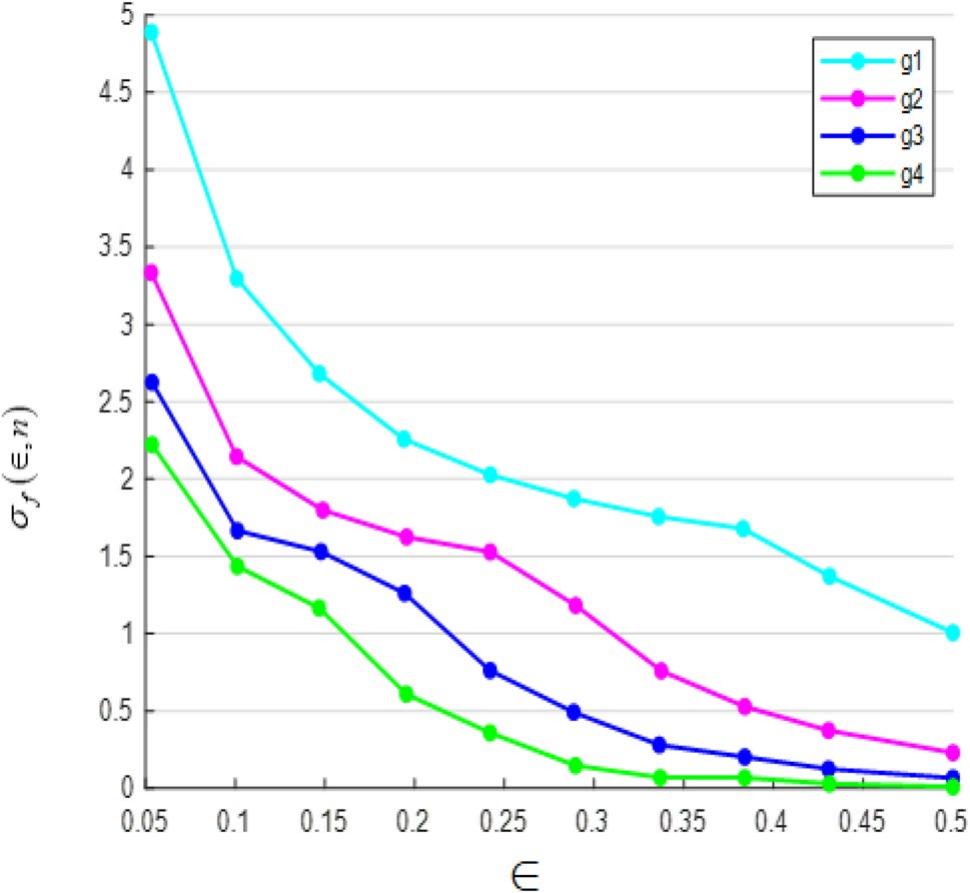
Ratio of theoretical accuracy and actual accuracy of the sign function approximation by the polynomials *g*_n_(*x*) on the interval *x* ∈ [є, 1].

Figures 9 and 10 illustrate the relationship between theoretical and actual accuracy in approximating the sign function using polynomials g_n_(x). Figure 9 presents the absolute difference between theoretical and actual accuracy across the interval x∈[є,1]. The results show that the gap between theoretical and actual accuracy is more significant for smaller values of є and decreases as є increases.

Figure 10, on the other hand, represents the ratio of theoretical accuracy to actual accuracy. The trends indicate that theoretical predictions consistently exceed actual accuracy, particularly for smaller є, highlighting the limitations of polynomial approximations in precisely capturing the behaviour of the sign function in this region.

Figures 7, 8, 9 and 10 measure the accuracy disparity between theoretical and real polynomial approximations. They confirm the stability and reliability of the polynomial-based operations implemented in the secure signal processing unit. This reliability is important for maintaining the security of the PUF-based authentication and aids in lessening unnecessary re-computation or errors, which all promote overall energy efficiency.

### Computation time analysis

**Fig. 11:**
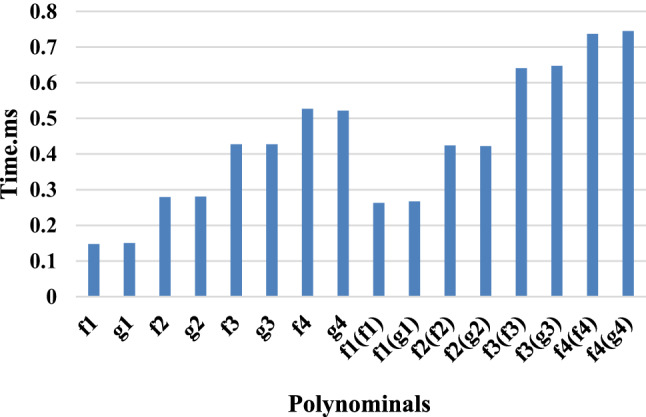
Computation time for the polynomials approximating the sign function.

The computation time expected for polynomial approximations was similarly surveyed. Figure 11 addresses the estimation time for different polynomial approximations, including the impact of polynomial degree on computational efficiency.

The examination shows that while additional serious level polynomials offer prevalent precision, they also achieve longer computation times. Consequently, a congruity among accuracy and computational efficiency ought to be pondered while picking polynomial approximations.

### Performance evaluation of BCEER protocol

In a comparative analysis of six routing techniques across networks ranging from 200 to 1000 nodes, BCEER consistently emerged as the top-performing solution. The evaluation encompassed various performance parameters, including energy consumption, throughput, network longevity, and routing overhead.

**Table 2:** Parameters of IoT enabled smart agriculture systems.

Parameters	Description	Values
Network Lifetime	Duration of system operation before maintenance	6 months
Energy Consumption per Node	Energy consumed by each sensor node	0.5 Joules per hour
Packet Delivery Ratio (PDR)	Ratio of successfully delivered packets	95%
Routing Overhead	Additional communication overhead due to routing	10%
Network Coverage	Percentage of agricultural area covered	90%
Latency and Response Time	Time taken for data transmission and feedback	2 s
Security and Privacy	Level of security measures in place	Homomorphic encryption, secure protocols

Table 2 summarizes the key parameters defining the operational characteristics of IoT-enabled smart agriculture systems, including network lifetime, energy consumption per node, packet delivery ratio, routing overhead, network coverage, latency, and security measures.

### Simulation setup

The diversion environment utilized 32 GB on an Intel i7 1065G7 Windows 11 working system to ensure efficient taking care of. MATLAB 2023a was used for making graphical depictions of essential limits, working with extensive assessment.

**Fig. 12:**
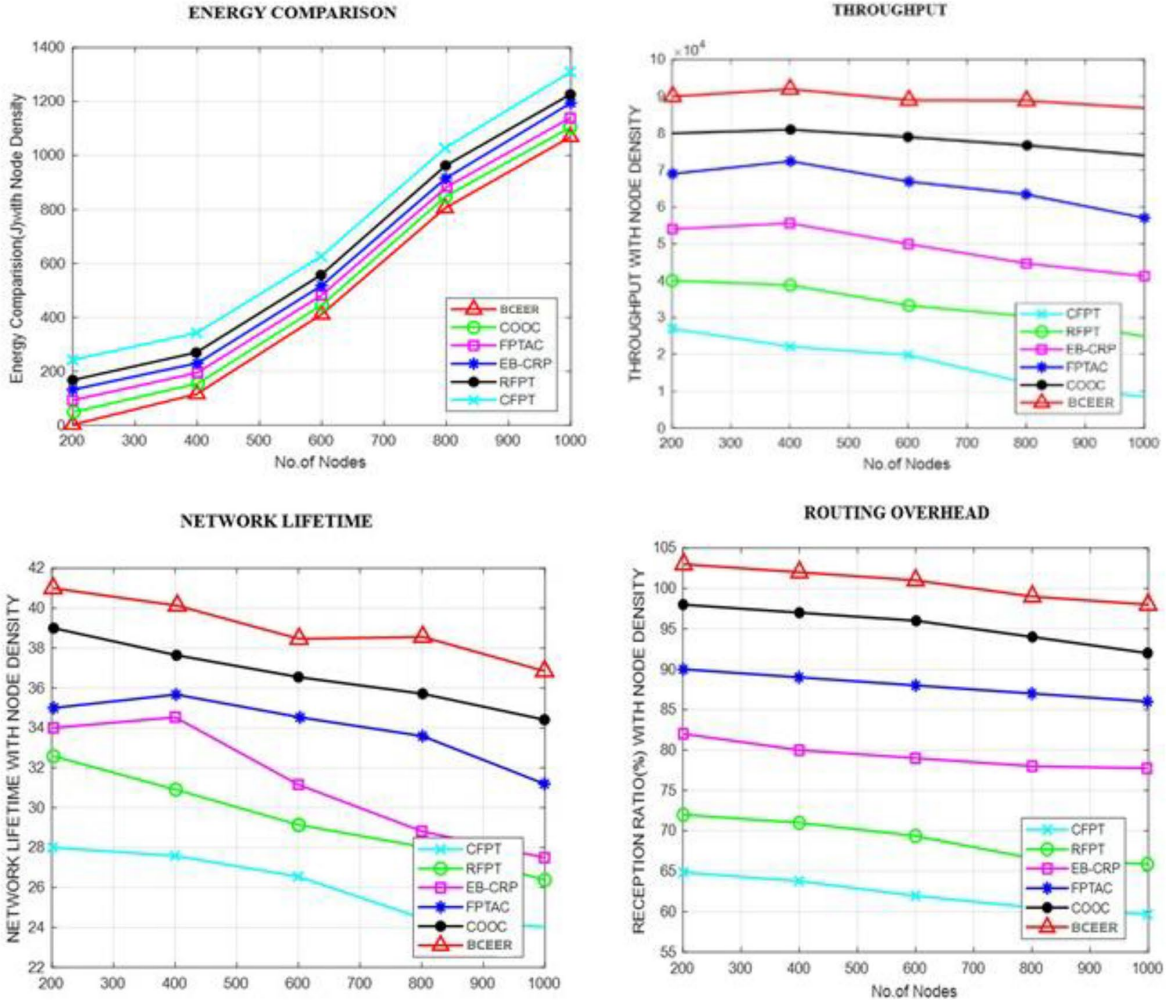
Energy comparison, throughput, network lifetime and routing overhead.

As per Fig. 12, the proposed BCEER outperforms the other methods such as Residual Energy-based Probabilistic Transmission (REPT), Energy Balanced Clustering and Routing Protocol (EB-CRP), Fuzzy-based Priority Transmission Aware Clustering (FPTAC), Clustering and Opportunistic Optimal Communication (COOC), Clustering-based Fuzzy Priority Transmission (CFPT) in terms of Energy comparison, throughput, network lifetime and routing overhead.

The appraisal results demand the ampleness and execution of the proposed approaches, highlighting their ability to overhaul energy viability, trustworthiness, and security inside IoT-enabled smart agriculture systems. The BCEER show shows pervasive execution across various estimations, featuring its propriety for certifiable plan in cultivating IoT associations. Moreover, the bumble assessment of polynomial approximations gives critical pieces of information into the trade-offs among precision and computational efficiency, assisting with the decision of legitimate gauge systems. The results, as a rule, feature the significance of synergizing sustainability through bleeding edge routing and authentication techniques in IoT-enabled agriculture systems.

## Conclusion

In frame, this assessment reveals a principal plan highlighted refreshing the security and energy reasonableness of IoT-engaged splendid green systems. Through faultlessly orchestrating Blended Clustering Based Energy-Compelling Controlling (BCEER) with Physical Unclonable Limit (PUF) based check, the proposed approach shows astonishing versatility to the cunning thought of IoT affiliations. Likewise, the getting of homomorphic encryption alongside as far as possible raises data legitimacy and social affair, giving overwhelming attestation against security shortcomings brand name in IoT natural frameworks. PUF-based affirmation adds an extra layer of watchman, interminably out planning the risks related with unapproved access attempts.

A fundamental evaluation of polynomial approximations for as far as possible remembers the fundamental control of polynomial affirmation for mess up minimization. Through a commensurate assessment, BCEER emerges as the unprecedented coordinating system across various evaluations in networks going from 200 to 1000 centre centres, overcoming rivals in energy sensibility, throughput, and controlling above update. While speculative approximations give goliath scraps of information, the verification of ensured troubles underlines the focal of sensible polynomial affirmation to ensure typical feasibility.

The organized arrangement, containing BCEER, PUF-based check, and precision polynomial approximations, addresses a key leap forward in refreshing the security, energy capability, and adaptability of splendid water structure systems. The encounters gathered from this study offer basic course for partners needing to grow practicality and execution in green IoT propels. As a promising procedure organized to address the creating circumstance of ordinary new development, the organized arrangement holds the monstrous potential to raise the overall efficiency of wise water structure systems, in this way making sensible green practices and working with redesigned crop yield and resource use.

## Data Availability

No datasets were generated or analysed during the current study.
